# Nuclear and Cytoplasmic hTERT, Tumor-Infiltrating Lymphocytes, and Telomere Elongation Leukocytes Are Independent Factors in the Response to Neoadjuvant Treatment in HER2-Enriched Breast Cancer

**DOI:** 10.3390/curroncol30040311

**Published:** 2023-04-07

**Authors:** Lucas Delmonico, José Bines, Cristina Moreira do Nascimento, Priscila Valverde Fernandes, Isabel de Souza Barbosa, Gabriel Brito Ribeiro, Bruno Henrique Rala de Paula, Rafaele Tavares Silvestre, Maria Helena Faria Ornellas, Gilda Alves, Claudia Lage

**Affiliations:** 1 Carlos Chagas Filho Institute of Biophysics, Federal University of Rio de Janeiro, Rio de Janeiro 21941-170, Brazil; 2 National Cancer Institute (INCA), Rio de Janeiro 20560-121, Brazil; 3 Pathology Division, National Cancer Institute (INCA), Rio de Janeiro 20220-400, Brazil; 4 Circulating Biomarkers Laboratory, Faculty of Medical Sciences, Department of General Pathology, Rio de Janeiro State University, Rio de Janeiro 22550-170, Brazil

**Keywords:** breast cancer, telomeres, hTERT, HER2-enriched, pathological complete response, telomere length, tumor-infiltrating lymphocytes, TERT promoter mutation

## Abstract

HER2-enriched tumors are responsible for 20% of breast tumors and have high rates of immune infiltrates in the tumor stroma that respond favorably to neoadjuvant chemotherapy. In the context of tumors, telomeres control cell death and prevent tumor cells from replicating discontinuously, leading to their immortalization. This study aimed to evaluate the presence of tumor-infiltrating lymphocytes, hTERT expression, hTERT promoter mutation, and leukocyte telomere length in HER2-enriched breast tumors. A total of 103 cases were evaluated, 19 with pathologic complete response. The TILs percentage was above ≥10 in 44 cases (43%) and significantly present in patients ≥50 years of age. hTERT staining positivity was mostly nuclear, significantly present in the non-pCR group, and associated with a lower survival rate. Leukocyte telomeres were elongated for HER2-enriched tumors, and in multivariate analysis, shortening was associated with an increased risk of death. Overall, our results show that the nuclear and cytoplasmic presence of hTERT may indicate a worse prognosis and that leukocyte telomere elongation is a protective factor.

## 1. Introduction

About 20% of breast tumors are defined by the overexpression or amplification of human epidermal growth factor receptor 2 (HER2). Using the immunohistochemical technique, these tumors are divided into luminal type B tumors (HER2 expression together with the expression of estrogen hormone receptors [ER] or progesterone [PgR]) which make up approximately 40–50% of HER2+ tumors; and HER2 enriched (HER2-E) tumors (~60% of HER2 tumors) that do not express hormone receptors (HR) [1]. Regarding their histological profile, HER2-E tumors present a higher grade and tumor staging at diagnosis and are clinically more aggressive. However, with the current anti-HER2 therapies, such as trastuzumab, pertuzumab, trastuzumab-emtansine (T-DM1), lapatinib, tucatinib, and trastuzumab deruxtecan, the survival of these patients has improved remarkably [2], with 30–40% surviving 8 years after the metastatic disease diagnosis [3].

Unlike normal breast tissues, where there is a significant immune aggregate absence, breast tumors may present high levels of immune infiltrates in the tumor stroma [4]. Most antigens present in breast cancer are self-proteins that can stimulate T cells and induce a regulatory immune response [5]. The relationship between the immune system and cancer is based on the level of cellular immunity promoting tumor growth, or also eradicating the disease hypothesis [6]. The so-called tumor-infiltrating lymphocytes (TILs) are present in 10%, 15%, and 20% of ER+ HER2−, HER2+, and HR-HER2− breast tumors, respectively [7]. Recent studies have demonstrated the prognostic and predictive role of TILs. In HER2+ breast cancer, elevated TILs at diagnosis result in a greater response to adjuvant trastuzumab treatment [8], and TILs are predictors of complete pathologic response (pCR) after neoadjuvant treatment [9]. Furthermore, it has been shown that the lymphocytes present in the bloodstream are directly related to tumor regression. Interestingly, it has been reported that the clonal expansion of T lymphocytes with the elongation of telomeres in the bloodstream has favored better responsiveness [10]. As a result of this mechanism, lymphocytes remain in the blood for longer, favoring the memory immune response against the tumor [10]. In this context, there is a link between the systemic immune response, the presence of TILs, and telomere activity. T lymphocytes with the elongation of their telomeres have been associated with tumor regression, unlike those with shortened telomeres [10,11]. Patients with a shorter relative lymphocyte telomere length (RLT) showed significantly poorer overall survival (OS) and progression-free survival (PFS) than patients with longer RLT [11]. 

Telomeres are repetitive DNA sequences (5′-TTAGGG-3′) located at the ends of chromosomes, essential for protecting their final portions against degradation, fusion, and end-to-end rearrangement [12]. In more aggressive breast tumors, such as luminal B, HER2-E, and basal, shortened telomeres have been associated with a worse prognosis [13]. Telomerase is the enzyme responsible for telomere maintenance and elongation, but it is not expressed in mature somatic cells. On the other hand, it is reactivated in most tumor cells responsible for the lack of cell cycle control that leads to tumor cell immortalization [14]. Interestingly, telomere shortening with telomerase reactivation is associated with worse OS in HER2+ breast tumors [15], as it is present in the most aggressive breast cancer cell lines [16]. The hTERT gene, which is responsible for encoding reverse transcriptase, is located at the chromosomal position 5p15.33 and has 15 introns and 16 exons, with exons 5 to 9 responsible for encoding reverse transcriptase [17,18]. From splicing, the gene can encode 22 isoforms, in addition to the fact that, in its promoter region, the numerous CpG islands act as ligands for transcriptional factors, reducing or accelerating the coding process [17,18,19]. Furthermore, the somatic mutations C228T and C250T present in the promoter region of the *hTERT* gene are prevalent in about 12% of solid tumors. They are responsible for telomerase reactivation, disease progression, and recurrence. Although rare in common forms of breast cancer [20], they have been reported at high frequencies in metaplastic and phyllode malignant breast tumors [21,22].

The objectives of this study were to evaluate the possible correlation between TILs, hTERT expression, *hTERT* promoter mutation (C228T and C250T mutations), and leukocyte telomere length (LTL) with the response to neoadjuvant chemotherapy based on anthracycline-taxane-trastuzumab or fluorouracil-anthracycline-taxane-trastuzumab regimen (AC-T/FAC-T) in a retrospective Brazilian cohort from HER2-E breast cancer patients.

## 2. Materials and Methods

### 2.1. Patients

Patients diagnosed with HR− (ER and PR score <1%) and HER2+ (3 or 2+ with FISH amplification) breast cancer that were treated with neoadjuvant therapy at the Instituto Nacional de Câncer (INCA) in Rio de Janeiro, Brazil between January 2010 and December 2015 were included. Patients received neoadjuvant chemotherapy with anthracycline plus cyclophosphamide, followed by paclitaxel plus trastuzumab neoadjuvant therapy at institutional-recommended doses and trastuzumab (adjuvant target therapy) for a total duration of one year. All correlations of the biomarkers studied here were based on responses to neoadjuvant chemo/targeted therapy. The Ethics Research Committee approved this study. Data on age, breast cancer family history (1st-degree relative), tumor type, immunohistochemical profile, size, nodal involvement, grade, and pathological complete response (pCR) were obtained from hospital records. All breast lesions obtained were formalin-fixed paraffin-embedded (FFPE) tissues. The histologic classification was graded according to the current (2012) World Health Organization criteria [23], and nuclear grade was defined as grades I to III, according to Elston and Ellis [24]. The histologic classification and the nuclear grading were performed by a medical pathologist (CMN).

### 2.2. Tumor Infiltrating Lymphocytes (TILs) Determination 

The Stromal TILs’ quantification was performed before neoadjuvant chemotherapy, according to the TILs Working Group recommendations [25], by counting all mononuclear cells in the stromal compartment within the invasive tumor borders and reporting them as a percentage value.

### 2.3. hTERT Immunohistochemistry

hTERT immunohistochemistry analyses were performed using FFPE before neoadjuvant chemotherapy. Briefly, 3-micron slices were deparaffinized and antigen retrieval was performed in a Trilogy Buffer (Cell Marque, Sigma-Aldrich, Rocklin, CA, USA), at 98 °C, using the steam process for 30 min. Endogenous peroxidases were blocked with a NovoLink Max Polymer Detection kit (Leica Microsystems, Wetzlar, Germany) for 5 min. Anti-TERT (Thermo Fisher— Monoclonal Antibody-2C4-Cat Number MA5-16034, 1:1500 dilution) antibody was incubated overnight at 4 °C. After incubation, the post-primary antibody and the polymer (Novolink, Newcastle upon Tyne, UK) were added and incubated for 30 min, rinsed, and exposed to a solution of diaminobenzidine for 3 min. Next, the samples were dehydrated with alcohol, cleared in xylene, and mounted. Negative controls were acquired using the same protocol described above, omitting the primary antibody. Immunoreactivity was determined based on the intensity and percentage of the staining of nuclear and cytoplasmatic tumor cells. For nuclear labeling, 100 tumor cell nuclei were evaluated. Tumors with <15% of nuclear immunoreactivity, were classified as negative, and those ≥15% were positive tumors. For cytoplasmic staining, the expression was quantified by stabilizing the score based on the positive cells’ percentage (0, <5%, 1, ≥5–35%, 2, ≥35–65%, and 3, ≥65–100%). The evaluation of counts was performed at 10X magnification by an experienced pathologist (CMN).

### 2.4. Qualitative Evaluation of hTR/hTERT and Splice Variants in Breast Cancer Tissues and Leukocytes plus Investigation of Mutations in the hTERT Promoter Region

The qualitative molecular evaluation of *hTR/hTERT* gene expression and the alternative *hTERT* gene (α+/β+) splicings in paraffin-embedded tissues and leukocytes were evaluated following the previously described methodologies [26,27]. In addition, the evaluation of mutation in the *hTERT* promoter region (C228T and C250T mutations) in breast tumor tissues was performed according to the methodology described by Trung et al. (2020) [28].

### 2.5. Leukocytes Telomere Length (LTL)

LTL from patients with HER2-E breast cancer was compared to leukocytes from a group of patients with HR+ breast tumors (*n* = 35) [29] and controls with no history (*n* = 89) or other conditions of breast pathologies (cohort from another study approved by the local ethics committee from the hospital of the Rio de Janeiro State University [UERJ/HUPE]). The evaluation of the LTL of the tumor groups and controls had an exploratory and comparative objective, in addition to determining the correlation of telomere shortening that is expected with age.

To maintain the exclusion of any red blood series from leukocytes archived at −20 °C, these were rapidly thawed at 37 °C and subjected to erythrocyte lysing solution (4 °C) (10 mmol/L Tris-HCl, 5 mmol/L MgCl2, and 10 mmol/L NaCl). The pellet was resuspended in a 600 μL lysis solution (10 mmol/L TRIS, 2 mmol/L EDTA, and 400 mmol/L NaCl) and 15 μL SDS 20%. The DNA extraction was performed using the Phenol–Chloroform method. DNA was quantified on the NanoDrop 1000 Spectrophotometer (Thermo Scientific, Wilmington, DE, USA) and Qubit dsDNA HS Assay Kit (Invitrogen, Waltham, MA, USA), according to the manufacturer’s protocol. Telomere length (TL) was measured using the real-time quantitative PCR method developed by Cawthon, which consists of determining the ratio of telomere repeat copy number (T) to single copy gene (S), T/S [30]. The single-copy gene used in this study was human β-globin (Hbg). The cycling conditions, primers, and reagents used in this study were as previously described [31]. The PCR runs were duplicated on a StepOnePlus Real-Time PCR System (Applied Biosystems).

### 2.6. Statistical Analysis

Contingency tables were used to assess the association between age, presence of TILs, nuclear and cytoplasmic hTERT staining, and pCR. For the purpose and evaluation of the statistical analysis of the correlation between variables, women were divided into age groups of <50 and ≥50 years old. The χ2 and Fisher’s exact tests were adopted to evaluate the statistical significance of the association between these variables. The Kruskal–Wallis non-parametric test was used to assess the significance of the difference between the telomere lengths of the evaluated groups. Linear regression was used to evaluate the correlation between age and telomere length between evaluated groups [11]. Survival distributions were estimated with the Kaplan–Meier method, and the significance of differences between survival rates was ascertained with the log-rank test. In addition, Cox regression (proportional hazards model) was used for multivariate analyses [11]. The survey data were processed in GraphPad, version 9.4.1. In all statistical tests, a 5% significance level was considered. Thus, statistically significant associations were considered to be identifiable in those whose *p*-value was <0.05.

## 3. Results

### 3.1. Clinical Data, pCR, and TILs

This study included 103 cases of HER2-E breast cancer. A total of 53% of patients were younger than 50 years of age (*n* = 55), and the remaining 48% (*n* = 47) were aged over 50 years. Regarding race, 51% (*n* = 53) were black and 33% (*n* = 34) were white. In total, 33 patients (32%) reported having a positive family history, with 12/33 (36%) being breast cancer. Regarding the tumor stage, all were locally advanced, predominantly stages IIIA (*n* = 38/103 [37%]) and IIIB (*n* = 36/103 [35%]), with infiltrating ductal carcinoma (IDC) encompassing 99% of the sample size. A total of 19 (19/103 [18%]) had pCR and TILs; <10 were present in 43/103 (42%) and ≥10 in 44/103 (43%) of the tumor specimens (Table 1).

### 3.2. hTERT Expression by Immunochemistry 

The nuclear and cytoplasmic labeling of hTERT could be evaluated in 81 cases. hTERT was predominantly expressed in the nucleus of 80% (*n* = 65/81) and with no or low expression (labeling 0 or 1) in the cytoplasm of 52% (*n* = 53/81) of the cases (Table 2).

**Figure 1:**
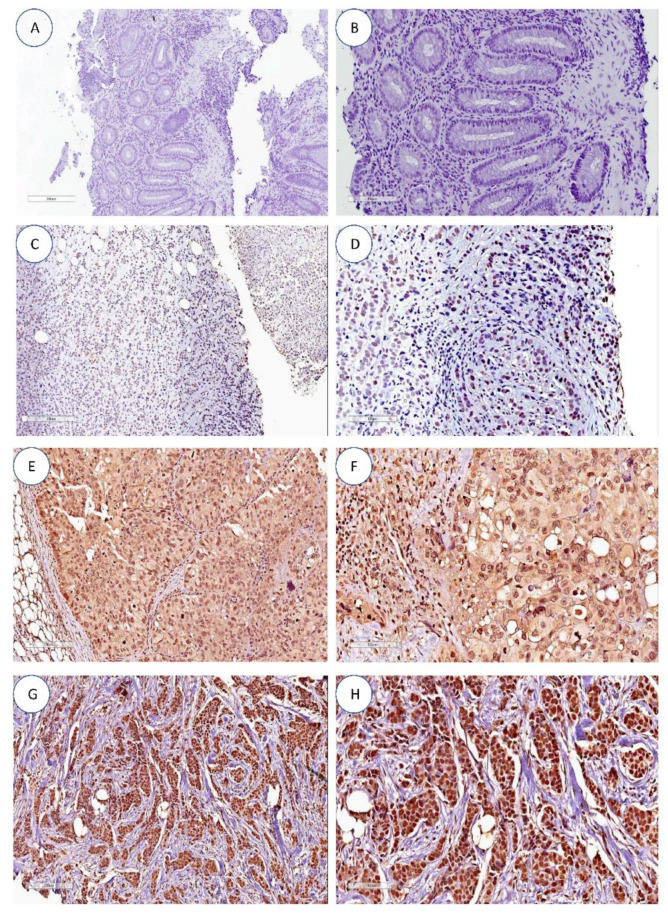
hTERT expression by immunohistochemistry. (**A**,**B**) Negative controls. (**C**,**D**) Absence of hTERT nuclear and cytoplasmic staining in infiltrating ductal breast carcinoma. (**E**,**F**) Predominant nuclear hTERT staining (grade 3) in infiltrating ductal breast carcinoma. (**G**,**H**) Diffuse cytoplasmatic and nuclear hTERT staining (grade 3) in infiltrating ductal breast carcinoma. All images are at 10× and 20× magnification.

hTERT expression by immunohistochemistry between the nucleus and cytoplasm was present in 39/81 (48%) cases, with 26/81 (32%) and 5/81 (6%) cases showing unique nuclear and cytoplasmic expression (Figure 1), respectively. Also, 11/81 (13%) showed negative cases for cytoplasm and nucleus expression (Table 3). These data were in agreement with the results of the evaluation of hTERT qualitative expression by PCR, where negative cases for hTERT expression were also negative in the expression evaluation by PCR (Section 3.4).

### 3.3. hTERT Expression by Immunochemistry and Correlation between the Clinical Variables 

The statistical evaluation of clinical data regarding age, pCR, TILs, and cytoplasmic and nuclear hTERT labeling are shown in Table 4. hTERT expression in the nuclear portion of tumor cells showed a significant relationship with non-pCR (*p* = 0.008). For the other evaluations, there was no statistically significant relationship (Table 4).

### 3.4. hTR/hTERT Transcripts Qualitative and hTERT Promoter Region Evaluation

The qualitative expression evaluation of *hTR/hTERT* genes was compatible with the molecular data previously presented, where 11/81 (13%) of the cases did not present any transcripts expression (Table 3). In other cases evaluated by immunohistochemistry (81/103 [79%]), even when the staining was exclusive to the cytoplasm or nucleus, the expression was present in the qualitative assessment and justified by the tumor total portion evaluation in the molecular analysis.

All 103 cases had *hTR/hTERT* expression present in the leukocytes. These data are consistent with those expected for blood cells, as the *hTR/hTERT* expression levels for these cells have already been described as independent of telomerase activity [32].

The *hTERT* splicings also identified failed qualitative and quantitative techniques [26,27]. The HL60 cell line, known to express the main *hTERT* splicings (TERT-2164 and TERT-2620), was used as a positive control and proved the reaction’s success. As these samples were preserved in paraffin and blood, stored for a prolonged period (>5 years), and the mRNA proved unstable, the analyses may have been unfeasible.

No mutations were found in the 103 cases for the *hTERT* promoter region (C228T and C250T).

### 3.5. Leukocyte Telomere Length 

**Figure 2:**
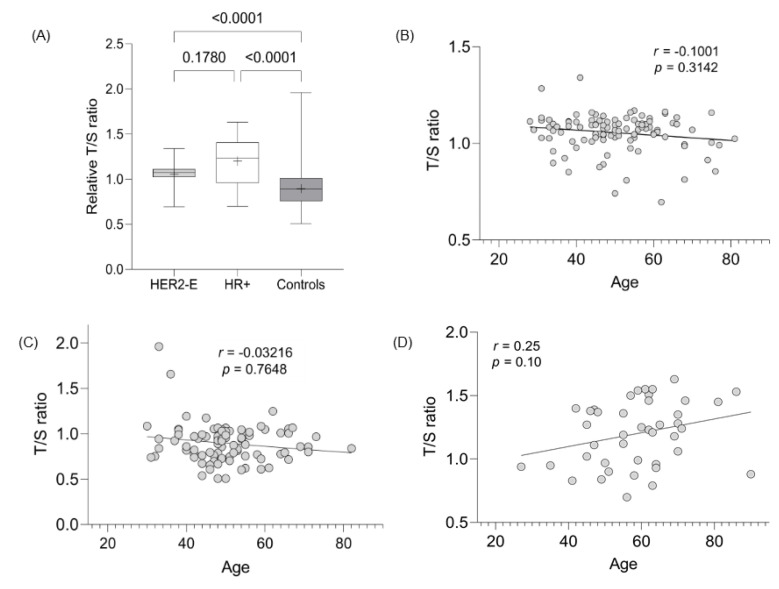
Evaluation of TL between groups and its correlation with age. (**A**) Significance TL assessment between breast and control tumor subtypes. (**B**) Correlation between age and LTL from HER2-E cases. (**C**) Correlation between age and LTL from controls. (**D**) Correlation between age and LTL from HR+ cases.

The LTL assessment was evaluated in 103, 35, and 89 cases of HER2-E, HR+, and controls, respectively. The mean LTL ratio (T/S) for HER2-E, HR+, and controls was 1.06 (SD 0.10), 1.22 (SD 0.26), and 0.90 (SD 0.21), respectively, demonstrating statistically significant values between the tumor groups evaluated with the control group (*p* < 0.0001) (Figure 2A). The difference in TL between the HER2-E and RH+ groups did not present a statistically significant value (*p* = 0.17). Since age and telomeres are dependent variables, linear regression evaluation showed a strong correlation between the decrease in TL with age for HER2-E cancer cases and controls (Figure 2B,C, respectively), but not for HR+ tumors (Figure 2D).

### 3.6. Leukocyte Telomere Length in HER2-E Breast Cancer and Correlation with Other Variables

Regarding the other variables, the nuclear and cytoplasmic hTERT positivity, TILs presence, and pCR were not statistically significant with telomere shortening in HER2-E tumors (Table 5). In the control group, telomere shortening in relation to increasing age was significant (*p* = 0.003) (Table 5).

### 3.7. Survival Progression-Free Survival Assessment of HER2-E Cases

**Figure 3:**
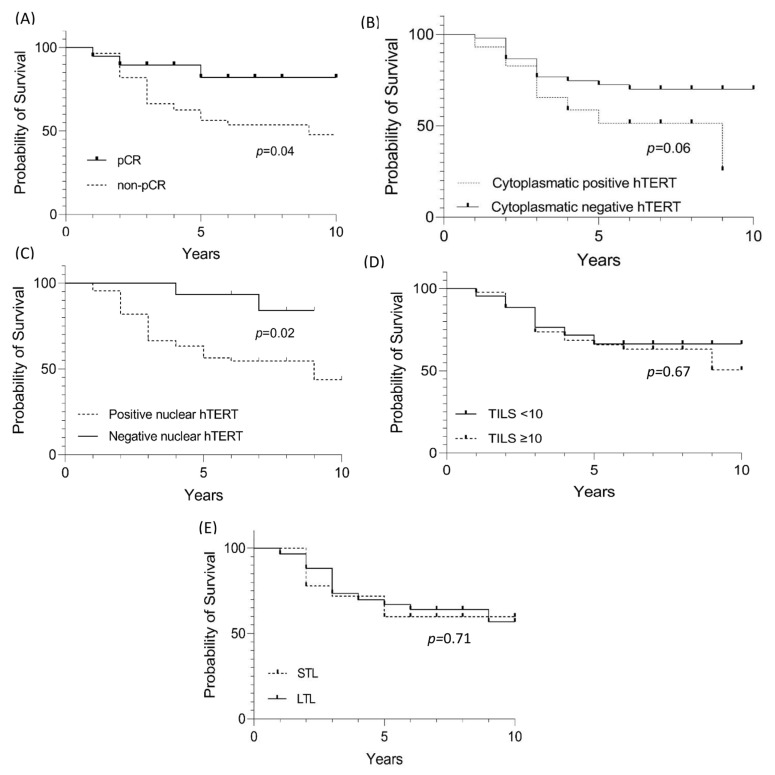
Survival evaluation for HER-E cases with pCR, hTERT cytoplasmic and nuclear staining, TILs, and LTL. (**A**) Evaluation of significant value in the survival of HER-2 cases with pCR. (**B**) Evaluation of significant value in the survival of HER-2 cases with hTERT cytoplasmic staining. (**C**) Evaluation of significant value in the survival of HER-2 cases with hTERT nuclear staining. (**D**) Evaluation of significant value in the survival of HER-2 cases with TILs. (**E**) Evaluation of significant value in the survival of HER-2 cases with shortening (STL—short telomere length) and lengthening (LTL—long telomere length) of leukocyte telomeres.

Patients with tumors pCR had a better clinical outcome compared to those with a non-pCR (*p* = 0.04) (Figure 3A). A total of 26 patients (70%) belonging to the non-pCR group died before the initial 3 years of follow-up, while for the 19 cases with pCR, there were only 3 deaths, two before 3 years and the last one after five years.

For progression-free survival, cytoplasmic and nuclear hTERT expression was statistically significant and associated with worse survival (Figure 3B–E). However, multivariate analysis (Cox regression) revealed that leukocyte telomere shortening increases the risk of death with significant *p* (Table 6).

## 4. Discussion

Positive HER2 and triple-negative are described as the breast tumors with the highest TILs percentage, so they respond better to neoadjuvant therapy [33,34]. Here, we found that TILs were uniformly distributed among HER2-E tumors, with 43/103 (42%) and 44/103 (43%) cases having a percentage of TILs that was less than 10%, and above or equal to 10%, respectively. Interestingly, in patients aged 50 years or older, the presence of TILs, although not statistically significant (*p* = 0.31), was mainly present in this group (29/54 [54%]) (Table 4). These data regarding the increase in TILs with age are contradictory to the findings by Belkacem et al. (2021) [35] and Takada et al. (2022) [36], who describe a reduction in TILs presence in HER2-positive tumors; however, although the first study described this finding, it did not find a significant value between the variables, and the second borderline statistical value of *p* = 0.04. The TILs’ benefit in the response rate to neoadjuvant therapy has already been reported in large experimental cohorts, but it is controversial when segregated between different tumor subtypes. In a study by Hamy et al. (2019) [33], TILs levels were higher in cases that reached pCR (*p* = 0.001). When tumors were stratified, this association by multivariate analysis was seen only for triple-negative tumors (luminal *p* = 0.058, triple negative *p* < 0.001, and HER2 *p* = 0.341). Furthermore, when evaluating the TILs percentage in the tissues of these same cases obtained from surgery, they tend to decrease in HER2 (*p* < 0.001) tumors with pCR. In the study by Denkert et al. [34], the TILs presence in HER2 tumors contributed to pCR increase and consequently increased OS rates in these tumor subtypes. Due to the number of cases evaluated here and the uniform distribution of TILs, the association between pCR and TILs is limiting; however, 19 (18%) cases achieved pCR and the remaining 84 (82%) cases showed partial response to treatment, showing that pCR is a well-established surrogate marker for clinical outcomes, especially for HER2 and triple-negative breast tumors [37,38].

With each cell division, telomeres shorten due to the loss of their repetitive sequences, reaching a critical point where the cell loses its ability to divide and entering the senescence phase. However, in tumor cells, telomeres tend to reveal an atypical behavior with telomerase reactivation, the enzyme responsible for telomere regeneration, and integrity, consequently leading to cell immortalization [10,12,13,14,15,16]. In this context, relative TL has been identified as a potential cancer molecular biomarker based on the fact that tumor stage, severity, recurrence, and overall patient survival data showed that shortened telomeres are present in tumors with a worse prognosis [13,16,39]. Interestingly, TL data are not concordant when comparing tumors and blood (leukocytes). The tumor TL evaluation was not evaluated in this study due to material quality limitations. However, the qualitative evaluation of hTERT expression was compatible with the findings achieved via immunohistochemistry, with 11/103 (11%) cases being negative for hTERT expression (Table 3). Data from the TL evaluation in different subtypes of tumor cell lines revealed that cell lines of the luminal subtype (MCF7) had the greatest TL, followed by HER2-E (SKBR3), and triple-negative cell lines (MDA-MB-231), and also demonstrated that the cell lineages with greater invasiveness had shortened telomeres compared to those with less invasiveness [16]. These data corroborate the evaluation in breast tumor tissues after neoadjuvant therapy, where telomere shortening was associated with more advanced tumors (*p* = 0.030) and lymph node involvement (*p* = 0.031). Increased hTERT expression was associated with HR- tumors (*p* = 0.039). Thus, triple-negative tumors had shorter telomeres than other subtypes. Furthermore, telomere shortening was associated with lower DFS (*p* = 0.0076) and OS (*p* = 0.05) [15].

In this study, 81 cases were evaluated for hTERT labeling in the nucleus and cytoplasm. The marking found was predominantly nuclear in 26/81 (32%) and only 6% (*N* = 5/81) of cases with cytoplasmic labeling (Table 3). On the other hand, concomitant labeling between the nucleus and cytoplasm was found in 39/81 of cases, representing almost half of the evaluation (48%). Comparative evaluation with other studies of hTERT nuclear and cytoplasmic expression in breast tumors is limited. However, the immunofluorescent hTERT labeling in breast tumor cell lines HER2-E (SKBR3) shows that, in this tumor type, the labeling is predominantly nuclear, contrary to other tumor subtypes, which are more heterogeneous [16]. In this study, we evaluated hTERT nuclear and cytoplasmic expression, the first being associated with nonPCR (*p* = 0.008) (Table 4), consequently representing a lower survival compared to those cases without nuclear labeling (*p* = 0.02) (Figure 3C). These data are complementary to the findings of Uno et al. (2022) [40], given that the hTERT nuclear expression associated with HER2+ tumors was (*p* = 0.00159) and not found in HER2-E tumors. Furthermore, when cytoplasmic hTERT labeling was present in HER2 tumors, it was associated with resistance to systemic therapy (r = −0.593). Such an association cannot be shown, given the limited cases evaluable. Interestingly, in other tumor types, nuclear and cytoplasmic staining differ. In two cohorts, regarding lung cancer and lung cancer associated with idiopathic fibrosis, hTERT expression showed intense nuclear staining in the group of lung tumors associated with idiopathic fibrosis (*p* = 0.016). In contrast, cytoplasmic staining was not significant (*p* = 0.46) [41]. However, in hepatocellular carcinomas, the marking was predominantly and exclusively cytoplasmic in 86/135 (64%), while only 3/135 (2.2%) were associated with nuclear marking. Furthermore, in these cases cytoplasmic labeling was associated with the presence of hepatitis B surface antigen (*p* = 0.007) and poor cell differentiation (*p* = 0.043) [42].

Of the 103 cases evaluated, none presented mutations in the promoter region of the *hTERT* gene (C228T and C250T). In metaplastic breast tumors, known for their complex genomics and aggressive histological subtype, rates can reach 15% [22]. However, the absence of h*TERT* promoter hotspot mutations (C228T and C250T) is consistent with previous studies, since in the common forms of breast tumors, the rate of mutations is less than 1% [43]. When present in tumors, these mutations can stimulate telomerase reactivation, favoring tumor progression [21,22].

LTL results are generally inverse to tumor assessment findings [44,45]. The LTL assessment was evaluated in the 103, 35, and 89 cases of HER2-E, HR+, and controls, respectively. The data presented here show telomere elongation in HER2-E and HR+ cases (*p* < 0.001 for both) compared to controls, but do not differ from each other (*p* = 0.17) (Figure 2A). Furthermore, the correlation of age with telomere shortening is present in HER2-E tumors and controls, contrary to HR+ cases (Figure 2A,B,D). No significant values were found when LTL data were correlated with the other variables (positive nuclear and cytoplasmic expression, presence of TILs, and pCR), as shown in Table 5. In other studies, telomere shortening was associated with worse OS, i.e., telomere shortening is a risk factor for toxicity after systemic treatment. Patients with breast tumors whose telomeres were deemed critically short (<3 kb) were shown to have greater episodes of paclitaxel toxicity (*p* < 0.001), unlike those with elongated telomeres [11].

Regarding prognosis, the shortening or lengthening of telomeres in leukocytes is still controversial, with telomeres being slightly elongated in HR+ tumors (as shown in this study) but without a significant value relationship [44]. On the other hand, among the most robust risk studies for cancer development and TL assessment (*n* = 26,540 evaluated individuals), telomere lengthening is a risk factor for cancer development (*p* < 0.001). However, elongation persistence is a protective factor against cancer and non-cancer mortality [45]. According to these data, although the comparison of survival between the lengthening and shortening groups was not significant (Figure 3E), when evaluating the risk among multiple variables (pCR, nuclear and cytoplasmic hTERT, TILs and LTL), telomere shortening of leukocytes increased the risk for death by 1.9 (*p* = 0.04) (Table 6). Furthermore, the relationship between telomere shortening in non-cancer cohorts exclusively and the risk of death has been evaluated by Arbeev et al. (2020) [46]. From a cohort of 3259 subjects and an 11-year follow-up, the authors stratified the 1525 deaths into three groups and showed the risk-to-mortality ratio for a 1 kilobase decrease in LTL of 1.28, 1.13, and 1 to 53 for deaths attributed to cardiovascular disease, cancer, and other factors, respectively.

In summary, our study shows that pCR is a good prognosis indicator in neoadjuvant therapy, confirming the findings of other studies [33,34,37,38]. hTERT nuclear labeling in HER2-E breast tumors indicates a worse survival rate. Although the hTERT cytoplasmic labeling here is inconclusive, its presence has been shown resistance to systemic therapy in HER2+ breast tumors [40]. In the same context, leukocyte telomere elongation in patients with HER2-E and HR+ breast tumors were significant when compared to controls and showed a higher risk of death in the multivariate analysis. A higher number of patients with different breast tumor subtypes is needed to validate the tumor/leukocyte TL and hTERT prognostic/predictive value in responses to neoadjuvant chemotherapy.

## Figures and Tables

**Table 1:** Patient and tumor characteristics.

	Patients *N* = 103 (%)
Characteristic	
Age, years	
Mean	50.3
SD	10.1
Cases ≤ 50	55 (53%)
Cases > 50 years	48 (47%)
Race *	
Brazilian White	34 (33%)
Brazilian Black/Brown *	53 (51%)
NI	16 (16%)
Menarche	
<12	22 (21%)
≥12	65 (63%)
NI	16 (16%)
Alcoholism Intake *	
Yes	9 (9%)
No	78 (76%)
NI	16 (15%)
Tobacco use *	
Yes	17 (17%)
No	60 (58%)
Ex-tagagist	10 (10%)
NI	16 (15%)
Familial history	
Yes **	33 (32%)
Second Cancer	2 (2%)
No	52 (50%)
NI	16 (15%)
Histopathologic Classification	
ILC	1 (1%)
IDC	102 (99%)
Stage Classification	
IIB	25 (24%)
IIIA	38 (37%)
IIIB	36 (35%)
IIIC	4 (4%)
Pathologic complete response	
Yes	19 (18%)
No	84 (82%)
TILs	
<10	43 (42%)
≥10	44 (43%)
NI	16 (15%)

* self-reported data obtained from medical records. ** considering family history only first-degree relatives. NI—not informed or not possible to be evaluated.

**Table 2:** Nuclear and cytoplasmic labeling of hTERT.

Nuclear hTERT	Patients (*N* = 103)
Yes	65 (63%)
No	16 (16%)
NI	22 (21%)
Citoplasmatic hTERT	
0, 1	53 (52%)
2, 3	28 (27%)
NI	22 (21%)

NI—Unable to be analyzed due to the absence of tumor material in the analyzed slides.

**Table 3:** Exclusive and concomitant expression detailing of hTERT between nucleus and cytoplasm.

			Nuclear and Cytoplasmatic hTERT		
	Patients (*N* = 81) (%)	pCR *N* = 15 (%)	Relapse (*N* = 14) (%)	TILs < 10 (*N* = 7) (%)	TILs ≥ 10 (*N* = 7) (%)
Nucleus and cytoplasm	39 (48%)	5 (33%)	5 (36%)	3 (43%)	2 (27%)
Nucleus	26 (32%)	3 (20%)	5 (36%)	3 (43%)	2 (27%)
Cytoplasm	5 (6%)	0	0	0	0
No expression	11 (13%)	7 (47%)	4 (28%)	1 (14%)	3 (43%)
Total	81	15	14	7	7

**Table 4:** Significance assessment between hTERT expression and the clinical variables.

Nuclear hTERT	Age		*p*-Value	
	<50 (*N* = 51)	≥50 (*N* = 52)		
Negative (*N* = 16)	10 (20%)	6 (12%)	0.27	
Positive (*N* = 65)	30 (59%)	35 (67%)		
NA (*N* = 22)	11 (21%)	11 (21%)		
Total	51	52		
Cytoplasmatic hTERT	Age		*p*-value	
	<50 (*N* = 50)	≥50 (*N* = 53)		
Grade 0, 1 (*N* = 53)	24 (48%)	29 (55%)	0.49	
Grade 2, 3 (*N* = 28)	15 (30%)	13 (24%)		
NA (*N* = 22)	11 (22%)	11 (21%)		
Total	50	53		
pCR	Age		*p*-value	
	<50 (*N* = 50)	≥50 (*N* = 53)		
pCR (*N* = 19)	7 (14%)	12 (23%)	0.31	
nonpCR (*N* = 84)	43 (86%)	41 (77%)		
Total	50	53		
TILs	Age		*p*-value	
	<50 (*N* = 59)	≥50 (*N* = 54)		
<10 (*N* = 43)	26 (44%)	17 (31%)	0.31	
≥10 (*N* = 44)	15 (25%)	29 (54%)		
NA (*N* = 16)	8 (31%)	8 (15%)		
Total	49	54		
Nuclear hTERT	pCR		*p*-value	
	pCR (*N* = 19)	nonPCR (*N* = 84)		
Negative	7 (37%)	9 (11%)	0.008	
Positive	8 (42%)	57 (68%)		
NA	4 (21%)	18 (21%)		
Total	19	84		
Cytoplasmatic hTERT	pCR		*p*-value	
	pCR (*N* = 19)	nonPCR (*N* = 84)		
Grade 0, 1 (*N* = 53)	13 (68%)	40 (48%)	0.07	
Grade 2, 3 (*N* = 28)	2 (10%)	26 (31%)		
NA (*N* = 22)	4 (22%)	18 (21%)		
Total	19	84		
TILs	pCR		*p*-value	
	pCR (*N* = 19)	nonPCR (*N* = 84)		
<10 (*N* = 43)	7 (37%)	36 (43%)	0.59	
≥10 (*N* = 44)	10 (53%)	34 (40%)		
NA (*N* = 16)	2 (10%)	14 (17%)		
Total	19	84		
TILs	Nuclear hTERT		NA (*N* = 22)	*p*-value
	Negative (*N* = 16)	Positive (*N* = 65)		
<10 (*N* = 36)	8 (50%)	28 (43%)	7 (32%)	0.76
≥10 (*N* = 34)	6 (37%)	28 (43%)	10 (45%)	
NA (*N* = 11)	2 (13%)	9 (14%)	5 (23%)	
Total	16	65	22	
TILs	Cytoplasmatic hTERT		NA (*N* = 22)	*p*-value
	Grade 0, 1 (*N* = 53)	Grade 2, 3 (*N* = 28)		
<10	21 (40%)	14 (50%)	7 (32%)	0.45
≥10	25 (47%)	10 (36%)	10 (45%)	
NA	7 (13%)	4 (14%)	5 (23%)	
Total	53	28	22	

NA—not possible to be evaluated.

**Table 5:** Significant evaluation between the length of the telomeres with the clinical data.

TL (HER2-E) (*N* = 103)	Age			*p*-Value
	<50 (*N* = 47)	≥50 (*N* = 56)		
LT (*N* = 84)	37 (79%)	47 (84%)		0.61
ST (*N* = 19)	10 (21%)	9 (16%)		
Total	47	56		
TL (HR+) (*N* = 35)	Age			*p*-value
	<50 (*N* = 29)	≥50 (*N* = 6)		
LT (*N* = 25)	21 (72%)	4 (66%)		>0.99
ST (*N* = 10)	8 (28%)	2 (34%)		
Total	29	6		
TL (Controls) (*N* = 89)	Age			*p*-value
	<50 (*N* = 36)	≥50 (*N* = 53)		
LT (*N* = 33)	20 (55%)	13 (25%)		0.003
ST (*N* = 56)	16 (45%)	40 (75%)		
Total	36	53		
TL	Nuclear hTERT			*p*-value
	Negative (*N* = 16)	Positive (*N* = 65)	NA (*N* = 22)	
LT (*n* = 84)	15 (94%)	50 (77%)	19 (86%)	>0.99
ST (*n* = 19)	1 (6%)	15 (23%)	3 (14%)	
Total	16	65	22	
TL	Cytoplasmatic hTERT			*p*-value
	Grade 0, 1 (*N* = 53)	Grade 2, 3 (*N* = 28)	NA (*N* = 22)	
LT (*n* = 84)	42 (79%)	23 (82%)	19 (86%)	>0.99
ST (*n* = 19)	11 (21%)	5 (18%)	3 (14%)	
Total	53	28	22	
TL	TILs			*p*-value
	<10 (*N* = 42)	≥10 (*N* = 44)	NA (*N* = 17)	
LT (*n* = 84)	36 (86%)	35 (80%)	13 (76%)	0.57
ST (*n* = 19)	6 (14%)	9 (20%)	4 (24%)	
Total	42	44	17	
TL	pCR			*p*-value
	pCR (*N* = 19)	nonPCR (*N* = 84)		
LT (*n* = 84)	16 (84%)	68 (81%)		>0.99
ST (*n* = 19)	3 (16%)	16 (19%)		
Total	19	84		

TL, telomere length; LT, long telomere; ST, short length.

**Table 6:** Multivariate Cox regression.

Variable	Hazard Ratio/Estimate	|Z|	*p* Value	95% CI (Profile Likelihood)
pCR [No]	2.34	1.50	0.133	0.8557 to 8.296
Nuclear hTERT [Yes]	0.67	0.89	0.376	0.2870 to 1.755
Cytoplasmatic hTERT [Yes]	1.51	1.02	0.307	0.6676 to 3.275
LTL [Short]	3.39	1.98	0.048	1.172 to 14.33
TILs [<10]	0.55	1.55	0.121	0.2560 to 1.168

## Data Availability

The original anonymous dataset is available on request from the corresponding author.
